# Preparing for tenure at a research-intensive university

**DOI:** 10.1186/s12919-021-00221-8

**Published:** 2021-06-22

**Authors:** Michael Boyce, Renato J. Aguilera

**Affiliations:** 1 Department of Biochemistry, Duke University, Durham, NC 27710 USA; 2 Department of Biological Sciences, University of Texas, El Paso, TX 79968 USA

**Keywords:** Tenure, Biomedical science, Academia, Junior faculty, Research-intensive university, R1/R2 university

## Abstract

At research-intensive universities in the United States, eligible faculty must generally excel in research, teaching and service in order to receive tenure. To meet these high standards, junior faculty should begin planning for a strong tenure case from their first day on the job. Here, we provide practical information, commentary and advice on how biomedical faculty at research-intensive institutions can prepare strategically for a successful tenure review.

## Background

### Introduction

New tenure-track faculty members at research-intensive (R1 or R2) institutions [1] emerge from a competitive, months-long job search process, eager to begin their independent careers. At this early stage, the tenure process may seem far away – about 6 years at most institutions – but tenure-planning should begin as soon as possible. Of course, being a new faculty member comes with a steep learning curve. Nobel Laureate and former Howard Hughes Medical Institute President Tom Cech has likened this to getting a driver’s license: “All of a sudden you have all of this freedom to turn when you want to turn or to go straight when you want to go straight. On the other hand, you have to pay for the gas, and you’ve got some responsibility” (quoted in [2]). How to balance the day-to-day tasks of a brand-new faculty member with the long-term career-planning you need for your future? This special issue of *BMC Proceedings* contains other valuable articles on managing the specific and immediate challenges of your new job, such as how to set up a research lab [3]. Here, we focus on the longer-term strategic planning that you need to position yourself as a shoo-in tenure case.

## Main text

### Know the rules of the game

The crucial first step in preparing for tenure is to understand the processes and expectations at *your* institution, for *your* position. Tenure requirements at American R1/R2 institutions change over time. Generally speaking, faculty in the 1980s were expected to demonstrate excellence in research, teaching or service, but 20 years later, all three came to be essential, with research as the top priority in most cases [4]. Promotion and tenure criteria also vary by institution and can sometimes be frustratingly vague [4–7]. Ambiguous or general criteria can be good, insofar as they allow for flexible and holistic assessment of each candidate, but they can also be bewildering for new junior faculty, who crave clear expectations. First, get the official tenure and promotion guidance in writing from your institution. Often this is publicly available in a faculty handbook or similar document from the Provost or equivalent chief academic officer. Read the guidelines carefully and then discuss them with your network – your department chair, senior colleagues both inside and outside your department, recently tenured faculty and (ideally) colleagues who have served recently on appointment, promotion and tenure (APT) committees. Ask questions about anything that’s unclear and solicit advice about any “unwritten rules” that you should know, such as the relative weight placed on research, teaching and service at your institution. Many universities have Faculty Advancement or equivalent offices that offer workshops on tenure preparation for junior faculty – attend every year, to track any changes in expectations and to keep the goal on your radar. In short, know the rules. All of the advice that follows in this article is based on common themes among research-intensive US universities and our own experiences, but we stress that *all tenure processes are local*, and you must do your due diligence to learn the ropes and expectations at your own institution.

### Know the process

To get tenure, you’ll need to know what goes into a successful tenure dossier and how it is evaluated [8]. You can expect that your research, teaching and service accomplishments will be comprehensively assessed. For research, peer-reviewed publications (especially primary research, but also reviews and commentary) and grant funding are key. For teaching and mentoring, teaching philosophy, syllabi, course evaluations and other documentation are typically required, and lists of trainees in your lab and service on thesis committees are the norm. Service is generally given the least weight, but notable accomplishments in the outreach, science communication, public policy, mentoring, and diversity/equity/inclusion (DEI) realms are valued, provided they accompany strong research and teaching. We discuss each of these three areas in more detail below.

Learn the nuts and bolts of the tenure preparation process, including the timeline. For example, you may undergo a pre-tenure review after your third year or so. At this stage, you typically prepare the same documents as you would for tenure (but without external evaluation letters; see below) and members of the departmental or APT committee review this package. You’ll receive feedback, focusing on areas of weakness and offering advice for improvement. The committee may even recommend an early tenure process, in strong cases. Typically, a complete dossier will then be due to a department chair by the end of a faculty member’s fifth or sixth year on the job. It often gets a first-pass read by the chair and an ad hoc departmental committee, to generate a list of external reviewers whom the chair will invite to provide letters reviewing your tenure and promotion credentials. Once letters arrive, the departmental committee evaluates the case thoroughly and presents a recommendation to the department (usually tenured members only) for a discussion and vote. After that, the chair usually shepherds the case through next steps, including review by a school or college APT committee, a dean, a University-wide APT committee, the Provost and the President or Board of Trustees or Governors. Get clarity from your department chair about when your dossier documents will be due, how long the process takes after submission (typically 6–12 months), and whether you can provide updates along the way, such as late-breaking grants or accepted manuscripts.

In your first months on the job, learn what future milestones you’ll pass en route to preparing your tenure dossier. Will you have annual evaluations or reappointments by your chair, a committee or someone else? Will an official mentor or mentoring committee of senior faculty be appointed for you (or will you need to request one from your department head or organize it yourself)? Is there a mid-tenure review after 3 years or so? These examples illustrate structured ways to receive feedback on your progress even in early years, so seek out these opportunities if they’re not provided automatically. If your institution requires your CV in a particular format for the tenure dossier (which is common), ask for a template in your first month on the job and begin adding to it right away. To start, list everything, even seemingly small accomplishments or honors, such as invited talks at your own institution, 1 hour of service on a panel discussion or a blurb highlighting your recent publication in a different journal. Down the road, you can always trim some items from the CV if you like, but you can’t add what you can’t remember, so don’t rely on memory and keep the document updated in real-time. Another strategy is to keep a tenure folder in your desk drawer, containing information to be added to your dossier at a later date, such as seminar fliers of your talks, thank-you letters for services provided, invitations for paper and grant reviews, service on committees, etc. You may be surprised how much you’ve done after five or 6 years and how many things you would have forgotten if you didn’t keep this information in one place.

Mentoring and training undergraduate and graduate students are important not only for your research (see below), but also for your tenure evaluation at most institutions. As you prepare your tenure dossier, consider adding a mentorship statement that describes your training record [9]. For example, do you offer hands-on training in your lab yourself? Do you teach or participate in workshops, such as NIH Responsible Conduct in Research or Rigor and Reproducibility trainings? Do you teach manuscript- and grant-writing to mentees and others? Do you perform outreach to recruit students from historically excluded groups to your university? Be sure to mention if you’ve received formal mentorship training by your institution or a professional organization, such as the National Research Mentoring Network. Within your academic lifespan, you’ll train a significant number of students and it is therefore important to keep a record of these trainees and their whereabouts and accomplishments (publications, fellowships, invited talks/presentations, etc.). You can also track former trainees by asking them to create a LinkedIn or ResearchGate account while they are under your supervision. At one of our institutions (UTEP), we expect all undergraduates in our NIGMS-funded training programs to create LinkedIn accounts so that we can stay current on their achievements and facilitate our own grant progress reports, renewals and new submissions. If your trainees move on to great places to pursue their post-graduate careers, you should list that information in your CV or mentorship statement. This information will also be valuable for NIH training grant applications, which require you to list your mentorship accomplishment in your biosketch and trainee tables. Importantly, some institutions request letters from current or former trainees as part of the tenure review process, providing another good reason to be the best mentor you can.

When it’s time to write your tenure dossier documents, keep your multiple audiences in mind. Usually, a single research statement will be evaluated by a wide range of groups, including the colleagues in your department, experts in your specific field (i.e., the external letter-writers) and non-scientists, such as German or law or divinity faculty on a university-wide APT committee. Writing a document that’s accessible and exciting to all of these audiences is a challenge, and it pays to get lots of feedback on drafts from friends or colleagues in each of the above categories. In planning your document, it can help to begin with a broad and non-technical overview of your research and its significance in the field, to help orient non-scientist readers, and then work down to specifics and expert knowledge when showcasing your science for others in your specialty.

External letter-writers can be the most mystifying (or terrifying) audience for your dossier. They must typically be “arms-length” from you, meaning you’ve never trained or collaborated with them, yet also expert enough to evaluate your work, and senior or distinguished enough for their letters to carry weight at your institution. Although you may never learn who the letter-writers are, a department chair or senior colleague might ask you (often off the record) to suggest a few names of prospective referees. If you have this opportunity, name scientists who know and appreciate your work, whose letters will be credible and respected, and whom your department might not think of themselves. If your field has one prominent scientist who is a clear leader, your chair or committee is likely to know that and will invite her or him as a letter-writer themselves, so don’t waste a limited number of suggestions on obvious picks. If you choose to suggest referees from outside the US (e.g., to attest to your international research reputation), be sure that they are familiar with the standards and norms of American tenure letters. If necessary, consider requesting to block one or two scientists as letter-writers, if there are people in your field who are known bad actors or with whom you’ve had a professional conflict. But use this option sparingly and with ample justification, so as not to give the impression that you aren’t well-liked in your field. Whoever your external referees are, you want them to sing your praises and say that you would get tenure at their own institutions, a question they are usually asked to answer.

Finally, a word about *changing* the process: Institutions usually provide the opportunity to “stop the tenure clock” for specific reasons, such as family care obligations, temporary medical problems or other circumstances (including COVID-19). Choosing this option pushes back the date by which you will need to submit your dossier for tenure review, to account for reduced productivity during a defined period of time. To determine whether stopping the clock could be right for you, find and understand the applicable written policies from your institution and discuss them thoroughly with your chair, senior colleagues and other trusted advisors. It’s important to ask their thoughts on how clock stoppages are viewed at your institution and whether it would be a good tactic for your specific situation. Ultimately, of course, it’s your prerogative to decide.

### Research

As noted above, research is nearly always the primary tenure consideration for faculty at R1/R2 institutions [4, 10, 11]. A strong tenure case is built on a strong research program, with a track-record of exciting science, peer-reviewed publications and extramural grant support. Your research must be independent, meaning you should make it clearly distinct from that of your doctoral and postdoctoral advisors. Building on your prior experience and knowledge is good, of course, but you also have to demonstrate your identity as an innovative and independent investigator by moving in new research directions and making impactful contributions to your field. Another way to think about this is as building your own unique brand – become known as “the person who” (e.g., “She’s the leader in transcriptional control of NK cell development,” “He’s the guy who discovered the role of phase separation in subnuclear compartments”). Similarly, collaborating with other groups can be an excellent way to advance your science, but you should do it judiciously. For any collaborative project or publication, your individual and substantive contribution should be clear (e.g., to external letter-writers evaluating your research output), and you should never let an excessive number of collaborations result in scattershot science, such that your lab lacks its own cohesive research focus. For this reason, it might be necessary to include a detailed description of your contributions and those of your students and staff within the tenure dossier CV for each collaborative paper that you published during the tenure period. And when establishing new collaborations, it’s helpful to discuss goals, division of labor and even authorship on future publications up front, to align expectations and ensure the relationship goes smoothly.

When launching your lab, it’s usually wise to start more than one project, or at least pursue multiple independent approaches to one overarching theme, so that not all your eggs are in one basket. At the same time, of course, you must also avoid stretching yourself too thin in the process. It’s often prudent to have a mix of high-risk/high-reward and safer, meat-and-potatoes projects, to ensure that the lab will be productive while also aiming for impactful discoveries. You should be choosy in admitting graduate students, postdocs and staff to your group to work on these projects. Many new PIs are impatient to fill their empty labs with warm bodies and get the science started, but it pays in the long run to wait for the right rotation student or postdoc candidate, and not accept someone mediocre just for the sake of having personnel. The adage “You are your own best postdoc” frequently applies for the first few years of a new lab, when the PI often works at the bench. If your lab budget allows, consider spending money to save time. For example, if you’re hiring a technician, you may want to bring on a more expensive but very experienced candidate, who can work independently and perform managerial tasks in lab, freeing you up for your other responsibilities. This approach may be especially beneficial for brand new faculty, who must usually establish a good research training environment in order to attract top graduate students and postdocs. Similarly, pricey experiments (e.g., CRISPR screens or proteomics projects) can sometimes be a good early investment, as a way to generate preliminary data for future grants and to lay the groundwork for new projects later.

Assuming you’ve chosen a good team of graduate students, postdocs and technicians, you might also take on a few undergraduates to assist them. Many universities have training programs that select some of the best undergraduate students to work in research laboratories and generally provide student stipends and modest research funds. One avenue to recruit graduate students into your laboratory early in your career is to train a cadre of highly motivated undergraduates. In fact, one us (RJA) ran his laboratory with an excellent team of undergraduates, with two of them later joining the lab as Ph.D. students. These early training experiences can lead to the development of undergraduate research training grants that, once funded, allow many more students to participate in research. Participating in undergraduate training can be a rewarding experience, is often highly valued by colleagues and opens the door to research for students undecided about their career paths [12]. Experienced faculty find ways of linking up undergraduates with graduate and postdoctoral fellows to form highly functional research teams that can be an asset to any laboratory. The graduate and post-graduate mentors not only benefit from having an extra pair of hands to assist them in their projects, but also attain mentoring skills that will be valuable throughout their careers. Of course, it’s important to make sure that undergraduate training activities are viewed positively at your institution and department, and to be careful to accept only a manageable number of students who are committed to doing high-quality research, and not just looking to burnish their resumes.

Peer-reviewed publications are the coin of the realm and the primary metric used to judge research output during tenure evaluation and beyond. Be sure to know what your institution values most in publications. Do you need a certain number? Does the journal name matter? There’s a growing awareness that impact factors are a poor – and often harmful – way of judging research quality [13–15]. Nevertheless, citation-based metrics, like impact factor, are mentioned in the APT guidelines of many institutions [16, 17]. If your university assesses publications using this kind of quantitative metric, it behooves you to understand those rules and make decisions about manuscript submissions accordingly, perhaps in consultation with your chair or other trusted senior colleagues. In any event, target journals where peers and prospective letter-writers in your field will see your work, and always avoid predatory journals [18]. Submitting manuscripts as pre-prints (e.g., to bioRχiv) can help advertise your work, garner additional citations and even generate constructive criticism from the community [19–22]. Primary publications are the cornerstone of your research portfolio and should be your main focus, but influential or highly cited review or commentary articles in your field are also valuable scholarly contributions. Keep in mind that many journals accept unsolicited proposals or even complete manuscript submissions for review articles – if you have a great idea for a review that will fill a gap in the literature or reshape the way your field views a problem, you don’t need to wait to be invited by an editor before you write it.

### Funding

Funding is a crucial complement to research. Science costs money, and you’ll probably need grant revenue to bankroll your work beyond the start-up phase. In addition, extramural grant support for your lab shows that funding agencies and their peer review panels value your research, so their seal of approval will be viewed favorably from both scientific and financial perspectives by the people evaluating your tenure case. As a brand-new faculty member, it might be wise to apply for career awards from private foundations or similar sources. Your top priority must be to get your lab up and running, but career award applications are often short and straightforward, and can allow you to cash in again on the strong record of postdoctoral research that got you your faculty job in the first place. In the longer term, project-based grants from NIH, NSF, DoD, large foundations or other agencies are the standard way to keep a lab solvent. As always, know the tenure expectations at your institution – if you need a certain dollar amount in support, a certain percentage of your own salary paid from grants or a particular kind of award (e.g., NIH R01) for a strong tenure case, find that out early, so you can prepare far ahead of time for grant submissions, re-submissions and renewals.

To maximize your chances of funding success, take a strategic and multi-pronged approach. Grant-writing can be laborious and challenging, but try to embrace it as a way to crystalize your ideas and align your research questions and plans with your scientific goals. Gathering examples of successful grants from colleagues is a great way to begin. You can also seek out formal grant-writing training, such as from your institution’s faculty advancement office, professional societies [23] or popular commercial options like the Grant Writers’ Seminars and Workshops [24]. Ask well-funded colleagues who have served on review panels and take an interest in your success to read drafts of your proposals and provide candid feedback. Begin far, far in advance, so you have time to receive multiple rounds of feedback if necessary and to work with the staff and administrators at your institution on the budget and approval process, which can be lengthy. Success rates with funding agencies are never as high as we’d like, so prepare to take many shots on goal. Some applications may run up against bad luck (e.g., a low payline or a hostile reviewer), so submitting a number of applications to different agencies can be a good way to hedge your bets. Consider being flexible in how you approach your science, taking different angles on different grant applications to appeal to different sponsors, and follow up on the directions that get funded [25]. In all cases, of course, a grant application needs a solid hypothesis supported by compelling literature and/or preliminary data in order to have a chance at funding, so be sure your proposal will have those components before you commit the time to preparing it.

### Teaching

Teaching may be emphasized less than research in R1/R2 tenure cases [4, 10, 11], but a solid record of quality instruction is nevertheless essential. As always, learn the expectations for how much teaching you must do for a strong tenure dossier, and what evaluation metrics will be used. Many new biomedical faculty at research-intensive institutions are fresh off a postdoc where they had little or no teaching opportunities. Therefore, be proactive in seeking out instruction and mentoring to improve your teaching. You can sit in on senior colleagues’ classes and have them evaluate your own, to provide constructive criticism. Many universities have a Center for Teaching and Learning or equivalent. Taking advantage of the resources, workshops and expert advice from those groups can be invaluable for new faculty, and will demonstrate your proactive effort to capitalize on institutional support for strong teaching. Keep in mind that excellent teaching takes *a lot* of time, with several-fold more hours of preparation than actual classroom contact hours, especially for new faculty and/or new courses. Evolutionary biologist Joel McGlothlin has nicely captured this point, saying “I found that teaching your first class takes precisely all the time available” [26]. Be sure to schedule plenty of prep time. If possible, it’s also valuable to make your teaching synergize with your research or other interests – perhaps volunteering to teach a course that forces you to brush up on a field relevant to your own science, or helps you ground your own work in a big-picture context when you write a grant. McGlothlin writes that an “NSF program officer once told me that to write a good grant, I should try to imagine how my research could serve as an example in a textbook. This was so much easier to do after a couple of years teaching evolution to undergraduates” [26]. Teaching a course in your area of expertise is also a great way to convey your enthusiasm to your students and share personal anecdotes of learning about new discoveries at a conference or how breakthroughs in the field were made. Students appreciate hearing about this human side of science.

Looking ahead to the teaching section of your tenure dossier, find out early what kind of materials you’ll need to include. Teaching philosophy statements, sample syllabi and course evaluations are standard examples. Some universities have well-crafted and universally applied course evaluation systems, and others do not. To ensure proper documentation of your excellent teaching, ask your course directors about evaluations before you begin as an instructor in any class (even for a single guest lecture), and create an evaluation instrument – preferably in cooperation with experts at your Center for Teaching and Learning – if one wouldn’t otherwise be provided. Many tenure reviews also require peer evaluations of your teaching and will look for steady improvements over time, so teaching a new course every semester or quarter is not a good idea. Finally, it’s important to note that teaching evaluations frequently reflect bias against certain categories of instructors, such as women and/or *p*ersons *e*xcluded because of their *e*thnicity or *r*ace (PEER scientists [27, 28]). If you belong to these groups (or even if you don’t!), ask your chair or dean what steps are taken in your university’s APT process to mitigate these well-documented biases. In any event, always look past petty or egregious comments and focus on improvements based on constructive criticism.

### Service

Junior faculty can make important service contributions in many realms, such as DEI, public policy, science communication, outreach, curriculum development and academic administration. Service should always complement (and not detract from) strong research and teaching and would rarely be sufficient for tenure alone. However, a track-record of impactful service shows colleagues how you contribute meaningfully to the community of scholars in your institution and can be a valuable component of a complete dossier. Service opportunities exist at the department, school, university, national and international levels, so you’ll want to think strategically about which activities appeal to you the most, will help advance your tenure case and represent a manageable time commitment. Notably, scientific or professional societies can be both a tremendous support for junior faculty and a source of significant service and leadership opportunities beyond your own university [29].

As a junior faculty member, you’ll want to select your service obligations carefully, such that they align with your values, help your own career and don’t overburden you. Consider saying yes to (or even volunteering for) service opportunities that allow you to contribute to your community while benefitting yourself. One example may be serving on a graduate admissions committee or teaching first-year students, to boost the odds of recruiting excellent people to your lab. Similarly, serving on a seminar-planning committee might allow you to invite prominent scientists in your field who could become reviewers on your grants, papers or tenure dossier, and reviewing a manageable number of grants or manuscripts for funding agencies or journals might be useful experience for preparing your own submissions later. Regardless of your interests, be sure to say no – politely but firmly – to service invitations that would seriously detract from your research, teaching or work-life integration. Junior faculty are usually somewhat shielded from unreasonable service requests, but don’t hesitate to ask for help from your department chair or other mentors if too many demands are made on your time. In particular, women and PEER scientists are disproportionately burdened with service commitments [4, 30–34]. These requests can arise from good intentions, such as a desire to broaden representation on a committee or a review panel. Nevertheless, women and PEER faculty especially should be mindful of their time and say no to service obligations that would threaten their other work responsibilities. Find out from your chair, mentors or peers what a typical service obligation looks like for junior faculty at your institution, and don’t feel the need to exceed that, if doing so would overtax you.

### Organization and time management

Now that you know *what* you need for a tenure dossier, *how* can you plan to build a great one? An essential first step is to be organized – when it comes to planning for tenure, “[b] esides productivity, organization is your best friend” [8]. As suggested earlier, keep your university-formatted CV up to date, starting in your first few weeks on the job, so you’re sure to have complete records of your achievements. Save documentation of your work, too. When you’re finalizing your dossier, course evaluations from a seminar you taught 5 years prior might be impossible to recover, so collect complete and well-organized records as you go.

Time management is also key, in several senses. First, you want to make strategic decisions about how much time to apportion to research, teaching, service and other activities, based on your own preferences and your institution’s priorities. Balancing these interests is a challenge for many faculty [4, 35–37], so seek help from your chair, mentoring committees, junior faculty peers, Faculty Advancement offices or scientific societies when you need it. Time management is also critical for accomplishing specific tasks. A postdoc is typically expected only to perform experiments and related work (e.g., writing manuscripts), whereas a junior faculty member faces a huge range of tasks, posing new organizational challenges. Keep a calendar, preferably an electronic one that synchs across all the Internet devices you use. Plan far ahead for major tasks, like preparing a grant or submitting a manuscript. Most junior faculty find that these take far longer than anticipated, from working through multiple layers of budget preparation and institutional approval on a grant application, to wrestling with a journal’s web interface to upload manuscript documents and information in the right order. Try to stay ahead of the game by starting tasks early. Before agreeing to any new commitment (a collaboration, a manuscript review, a new committee membership), be sure to understand the expected time demand and be realistic with yourself about whether it fits well with your availability and priorities. Learning to say no gracefully is both a common challenge and an essential skill.

Some additional advice for fellow procrastinators: It’s helpful to make yourself write at least 1 day a week, even if it’s just for a few hours. Block time on your calendar and write a few paragraphs for a paper, the Specific Aims of a grant, a conference abstract draft, etc. Many PIs prefer to work at the bench versus in the office, but regular and dedicated writing time can help you get the tasks done.

Finally, be kind and reasonable with yourself as you learn the ropes and accept that some things may not get done, or not done quite the way you would ideally want. McGlothlin acknowledges that juggling too many work tasks can often trigger guilt for junior faculty, but it’s possible to come to terms with this:

It becomes hard to focus on getting any one thing done because of the weight of the to-do-list albatross around your neck. I wish I could say that I found some magical time management solution to balance tasks and get caught up, but I never did. What I did realize is that it’s possible to let go of the guilt. You can forgive yourself for not getting things done on time or done as well as you would like, or for prioritizing one task (sometimes the wrong one!) over another. Yes, I still apologize to others when I’m late or otherwise let them down, but I try to forgive myself, cut the albatross loose, and move on [26].

### Professional skills development

New tenure-track faculty at R1/R2 universities often have little formal training for many of their responsibilities, such as grant-writing, budgeting, hiring, managing, motivating and (perhaps) firing employees or students, teaching in a variety of formats, overseeing several research projects at once, complying with safety and ethics requirements on behalf of a group, and a wide range of academic service. Preparing a strong tenure case requires mastering most or all of these professional skills – a daunting task. One strategy is to seek out formal training for junior faculty, such as workshops offered through your institution’s Human Resources, Institutional Equity or Faculty Advancement offices. Some outside groups also offer well-regarded lab leadership courses, such as the American Society for Cell Biology (ASCB) and other professional societies, Cold Spring Harbor Laboratory and the European Molecular Biology Organization (some US training sites are available) [38–40]. There are also focused programs for new investigators to learn specific skills. For example, several scientific societies offer structured grant-writing programs to new PIs [41, 42]. The NIH Early Career Reviewer (ECR) program is another attractive option, allowing junior faculty to serve on NIH review panels with a reduced workload [43]. Junior faculty in the ECR program provide valuable service to the scientific community while also learning about grantsmanship and the inner workings of the review process, and networking with other panelists in the same field. Sometimes, gaining experience as a grant reviewer can be as simple as e-mailing the scientific review officer in charge of a relevant panel at NIH, NSF or another agency to volunteer your expertise on an ad hoc basis. Of course, learning grantsmanship or another professional skill by performing service is a valuable but time-consuming activity, and you’ll want to be sure that the cost-benefit analysis is in your favor before you agree. Beyond these approaches, junior faculty can also improve their skills through advice and mentoring by senior colleagues, through formal mentoring committees or individual conversations to discuss professional challenges and strategies. During the tenure preparation marathon, it pays to explore all of these avenues to hone your skills.

### Scientific recognition and networking

For a successful tenure review, your accomplishments must be appreciated by colleagues at your home institutions and beyond. We might all hope that “the research can speak for itself,” but the reality is that tenure (and all science) depends on doing high-quality work *and* ensuring that others know about it. Within your department, be sure that your colleagues understand and appreciate your research. If it’s highly interdisciplinary, in a brand-new field, or somehow different from most of the science in your unit, you may need to take extra care to educate your coworkers about its significance and value. Internal work-in-progress seminars on your campus and posters at department retreats (presented by you and/or your trainees) are a simple and collegial way to spread the word about your exciting projects. Outside your institution, seminars at other universities and talks at conferences are key ways of introducing yourself and your science to the community. It can be shrewd to pick one or two important conferences in your research area and attend them every year, presenting your work as often as possible, to raise awareness of your research and become a fixture in the professional network of the field. It’s also a good idea to build an attractive and informative lab web site, with your publications and research interests clearly listed. Many faculty also use social media, such as Twitter, as a tool to gather information (e.g., from journals, funding agencies and other scientists) and to advertise the accomplishments of themselves and their trainees, such as new pre-prints and papers, awards and grants. Consider applying for awards to recognize your achievements in research, teaching or service, or ask senior colleagues to nominate you when needed. A long list of awards is not typically a prerequisite for tenure, but some recognition beyond your institution doesn’t hurt.

Networking can be a tremendous help to junior faculty, by recruiting trainees, finding collaborators, meeting future grant or promotion evaluators, seeking help, raising awareness about your own accomplishments, and more. Networking may not come naturally to all scientists (or to everyone in any field), but it’s wise to learn to do it in a way that’s proactive and intentional, while remaining authentic to your own personality and style. You can begin at home, in your own department. Simple things like lingering for pizza after a seminar or joining a department happy hour or retreat to chat with colleagues and students can build collegiality and spark interesting scientific discussions. (To be clear, drinking is optional in all cases – networking at happy hour works just fine over a cup of coffee or glass of water!) Similar opportunities exist at conferences, when you visit other campuses for seminars, or when outside speakers visit you. As noted, it can be helpful to invite seminar speakers to your home institution who are likely to review your future manuscripts or grants, or write external letters for your tenure dossier (e.g., members of the NIH study section where your application will go). This strategy promotes your networking, by helping you establish good relationships with more senior scientists, and also aligns well with your research interests, because someone serving on a panel that would review your grants is likely to have similar scientific interests to your own, making them a natural choice for an interesting seminar speaker.

## Conclusions

### Wellness

Last but certainly not least, be sure to practice self-care and remain healthy and happy as you work towards tenure. To be sure, this can be a challenge. As McGlothlin notes about new faculty positions:

*For most people, this will … be their first experience leading a team. It will be the first time that there is no adviser to consult when there is a tough decision to make, which can be daunting at first. Unless you’re lucky enough to find students or postdocs right away, when you start you will be leading a team of one. This can be a huge adjustment for people used to being part of a large lab, as I had been as a grad student and a postdoc. The first year, when you’re working solo in your office or in an empty lab, can be incredibly isolating* [26].

It’s important to know that this is a very common feeling – it’s not just you! – and there are concrete things you can do when fear, doubt, impostor feelings or loneliness strikes. For starters, as Robert Bloch advises, “Don’t toil away in isolation” [44]. New faculty may hesitate to ask for help, worrying that it could make them look bad. On the contrary, seeking help when you need it is a sign of maturity, wisdom and proactive good management. Your department chair is often a good first stop. Chairs and other supervisors have usually invested a lot of time and resources in you as a new faculty member, and they genuinely want you to succeed. Get their advice and assistance when you need it (while remaining respectful of their time). It’s also helpful to cultivate good relationships with both senior colleagues in your department and/or field, and other junior faculty at your institution, so you can lean on them as sources of support and offer help in return when they ask. The most supportive and valuable colleagues are often those who take a sincere interest in your work and career, whether or not their science is closely aligned with yours, and give you their honest opinions, even when it’s tough love. Online communities, such as those offered by scientific societies or groups like New PI Slack, can also be excellent wellsprings of advice and moral support [45, 46]. And, of course, you’ll make mistakes along the way, as we all do. No one enjoys errors, but try to consider them as an inevitable part of the learning process and a sign of professional growth – however painful – and then move on. As Bloch advises new faculty, “Don’t fret about a decision once you’ve made it. You can usually correct a problem later, if you have to” [44].

Perhaps most importantly, try always to maintain a healthy work-life integration, however that is best defined for you. A tenure-track faculty position is an exciting and sometimes stressful career, but it’s only one element of life, and it has to be compatible with your physical and mental wellness, values, family and friends, religion or spirituality, and any other aspects of a fulfilling existence that matter to you. With some careful planning and help along the way, you can have a smooth arc towards tenure and beyond, while also keeping your balance in these other important realms of life.

## Data Availability

Not applicable.

## Abbreviations

ACT: Accomplishing Career Transitions
APT: Appointment, promotion and tenure
ASCB: American Society for Cell Biology
DEI: Diversity, equity and inclusion
DoD: Department of Defense
ECR: Early Career Reviewer
NIH: National Institutes of Health
NSF: National Science Foundation
PEER: Persons excluded because of their ethnicity or race
PI: Principal investigator
R1/R2: Research-intensive institution
